# Characterization of masculinity expressions and their influence on the participation of women in Mexican small-scale fisheries

**DOI:** 10.1007/s40152-022-00276-z

**Published:** 2022-07-21

**Authors:** Alejandra Salguero-Velázquez, Neyra Solano, Francisco J. Fernandez-Rivera Melo, Inés López-Ercilla, Jorge Torre

**Affiliations:** 1 Facultad de Estudios Superiores Iztacala, Universidad Nacional Autónoma de México, Mexico City, Mexico; 2 Comunidad Y Biodiversidad, A.C, Isla del Peruano 215, Lomas de Miramar, 85448 Guaymas, Sonora Mexico

**Keywords:** Gender equality, Masculinity, Identity transformation, Women in fisheries, Small-scale fisheries, Mexican fisheries

## Abstract

The objective of this study was to document and characterize the different expressions of masculinity and their influence on the participation of women in three small-scale fisheries. The fisheries operate in three priority regions for marine conservation: the northern Mexican Pacific, Gulf of California, and Mexican Caribbean. A qualitative investigation and virtual interviews with participants were conducted due to the lockdown measures of the COVID-19 pandemic. Information from 16 interviews (nine women and seven men) were integrated to this study. An analysis of the thematic content of the information was conducted. The results showed a relationship between being a man, being a fisher, and being a member of a fishing cooperative while maintaining a family. Four characterizations of masculinity emerged. In reluctant traditional masculinity, older men believed that their point of view was what mattered, and they did not accept the participation of women in fisheries. Men who fell under the category of flexible traditional masculinity accepted the participation of women in the working areas of the fisheries. Men associated with transitional masculinity were those who incorporated notions of gender equality and who were open to the participation of women in fisheries. Men connected with apprentice masculinity were mostly young people who depended on their supervisors to guide them. Apprentice masculinity may develop into traditional masculinity or move towards gender equality. The discussion incorporates an analysis of the benefits of changing masculinities regarding health, avoiding risks, and accepting the participation of women in fisheries.

**Supplementary Information:**

The online version contains supplementary material available at 10.1007/s40152-022-00276-z.

## Introduction

### Women in fisheries

According to the Food and Agriculture Organization of the United Nations (FAO), women constitute only 14% of the fisheries workforce involved in harvesting. However, when pre- and post-production activities are also considered, women make up half of fisheries workers worldwide (World Bank 2012; FAO 2020). Moreover, women in fisheries are also subject to a traditional gender-based division of labor in which they are often solely responsible for the home, domestic activities, and childcare, which further highlights current gender inequalities (Harper et al. 2013; Teh and Sumaila 2013, Solano et al. 2021). Women are not only relegated in fishing activities but also excluded in decision-making within fisheries management organizations like fishing cooperatives. Moreover, women do not usually become members of fishing cooperatives given that the activities they often participate in are not considered to directly contribute to the fisheries, except in cooperatives that are exclusively comprised of women. The advantages of being a member of a fishing cooperative include ownership rights (e.g., shared permits or concessions), guaranteed work, economic income, and the benefits derived from formal employment, such as health care and education (Solano et al. 2021). For example, in Mexico the internal structure of a fishing cooperative is democratic since all members have a voice and vote in decisions that affect the cooperative according to the principles of solidarity and the common good (DOF 1994). However, the organizational component of fishing cooperatives that is called into question is the gender bias that is present, as men have greater opportunities to participate in activities and receive benefits. In Mexico, this bias has been identified in the small-scale fisheries of the northern Mexican Pacific, Gulf of California, and Mesoamerican Reef System (Solano et al. 2021).

Resistance to the involvement of women in economic activities like fishing is widespread (ECLAC 2019; Perea-Blazquez and Flores-Palacios 2016), which is directly connected to the old dichotomy that places men in the public sphere and women in the private, domestic sphere. Gender cultures that are based on gender inequality affect living conditions, the conservation of natural resources, and sustainable development in both public and private spheres worldwide (López and Bradley 2017). Not only do gender cultures often violate human rights, but also they have direct impacts on physical and psychological well-being (Manjoo and McRaith 2011; Castañeda Camey et al. 2020). Gender cultures also continue to limit economic and educational opportunities for women, leading to the marginalization of women in decision-making (Castañeda Camey et al. 2020).

Gender inequalities associated with access to fisheries and participation in decision-making are largely due to the cultural construction that the fishing world belongs to men. This construction incorporates gender cultures in most of the organizational systems of fisheries like cooperatives, legitimizing male privilege based on a historically constructed idea that men are the ones who should fish and extract resources due to bodily attributes like physical strength, toughness, and endurance (Adkins 2010; Turgo 2014; Salguero and Alvarado 2017; Castañeda Camey et al. 2020).

Inequality is an obstacle to achieving the United Nations Sustainable Development Goals (SDGs), particularly SDG 1 (No Poverty), 5 (Gender Equality), and 14 (Life Below Water), and the Voluntary Guidelines for Securing Sustainable Small-Scale Fisheries in the Context of Food Security and Poverty Eradication (FAO 2015), which has undoubtedly resulted in a loss of many opportunities (Aguilar et al. 2015; ONU 2015; Coulthard et al. 2020). In 2019, the United Nations Educational, Scientific, and Cultural Organization (UNESCO) held the conference “Men, Masculinities and Gender Equality in Africa, the Caribbean and Latin America: Interregional Dialogues” in Mozambique, Africa. The outcomes of this conference emphasized that to meet the goals of the 2030 Agenda for Sustainable Development regarding the democratization of social relationships and gender equality, it is essential to work and collaborate with men (UNESCO 2019). Otherwise, in many spaces, women will continue to be undervalued or excluded in decision-making due to the inequality present within workplaces (WorldFish Center 2010).

### Masculinities in Mexico

The interest in masculinity studies continues to rise (Rivera Gómez and Rivera García 2016). These studies consider gender relationally and analyze the ways in which men participate in society (García Villanueva 2017). The existing consensus regarding male attributes is that the male gender is associated with productivity, strength, decision-making, and the tendency to hide or silence emotions, among others (García, 2005; Salguero and Alvarado, 2017; Siegelman et al. 2019). Connell and Messerschmidt (2005) argue that masculinity is not a “fixed entity embedded in the body or in the personal traits of individuals” but is rather a “configuration of practice” that is achieved in social contexts. Given that fishing is a highly social activity, different masculinities can be identified, with socially dominant traditional masculinities developing and persisting among fishers. However, masculinities that are defined by behaviors of respect and care towards themselves, their companions, and their families and that move away from the hegemonic model of masculinity are also present (Kaufman 1989; Coulthard et al. 2020).

The construction of traditional masculinity in the fishing sector is not only detrimental for women in that it prevents them from benefiting from the complete set of rights awarded to male fishers (Alonso-Población and Niehof 2019) but is also determinantal to men, hindering the relationships they establish with others, affecting their health, and encouraging substance use (Kaufman 1989; Kimmel 1997; García 2005; Tu-Anh et al. 2013; Turgo 2014; Coulthard et al. 2020). As such, it is necessary to analyze the health risks in men associated with the stress that comes from assuming the responsibility of holding a job and providing for a family, even when their partners contribute financially (Figueroa and Nájera-Aguirre 2015). Turgo (2014) considers that being a fisher, with all the dangers and challenges that accompany the activity, could well be the most powerful expression of hegemonic masculinity in a fishing community. Siles et al. (2019) refer to the widely generalized culture of toxic masculinity that is present in fishing communities, which not only damages the lives of men, but, as Ratner et al. (2014) suggests, continues to legitimize and preserve inequality when power is exercised in fisheries.

Masculinity is dynamic and constantly changing, and documented evidence of its transformations is available. To account for these changes among traditional characterizations of masculinity, it is necessary to view men as being subject to gender within specific social practices (Connell 1995; Núñez 2017) and consider the construction of male identities within contexts. A diversity of configurations exists among these constructions, but at the same time, particular characteristics within each configuration may be identified given that male identity is dynamic, contextual, relational, and tied to a specific time and place.

Recent studies have highlighted a possible nuanced change in fishing masculinities (Pini and Conway 2017; Gustavsson and Riley 2018, 2019; Coulthard et al. 2020). For example, Gustavsson and Riley (2019) report that changes in fishing masculinities are associated with changes in family life and income, with new practices being incorporated into the older and traditional aspects of fishing masculinity. These new forms commonly intermingle with traditional aspects of masculinity but do not replace them (Pini and Conway 2017).

Constructing and fostering a positive and non-violent version of masculinity in men requires specific knowledge, skills, advice, and support from peers. Not all men legitimize gender inequality, nor do they all display traditional hegemonic masculinities (Filteau 2014). In this sense, men display gender forms that are based on the contexts in which they developed. Some men develop new and alternative forms of non-hegemonic masculinities while still expressing opinions within their local communities that can be authoritarian. Therefore, it is important to recognize the situational components of gender and that masculinities are not static but dynamic (Hopkins and Gorman-Murray 2014), and it is essential to avoid adopting generalized constructions of fishing masculinities. According to Gustavsson and Riley (2019), recognizing that a single hegemonic fishing masculinity does not exist and that masculinities depend on their geographic, cultural, and temporal contexts is necessary to understand how masculinities can be redefined and reworked over time by sociocultural and structural changes in fishery industry organizations.

Changes in gender relations and masculinities are closely related to the commitments established in the International Conference on Population and Development and the World Conferences on Women. These conferences have marked an important turning point in the global agenda. Although much work remains, the conditions under which men and women participate in society have been called into question and analyzed. Unequal gender norms have highlighted that men have greater access and control over resources, such as forests, agriculture, water, and fisheries, than women (Castañeda Camey et al. 2020). Moreover, the inequalities that women face are due to engrained social disadvantages (Kabeer 1998).

Fishing is often the principal activity in coastal communities, although these coastal areas stand out for their diverse economic activities. The fishing sector continues to be dominated by men, with extractive activities considered to be traditionally male pursuits. This begs an important question: What happens when women want to participate in fisheries? Specifically, how do the men working in fisheries view the participation of women, especially the men who have been working in fisheries for decades?

In this context, the objective of this study was to characterize the expressions of masculinity and their influence on the participation of women in fisheries in three marine ecosystems of Mexico: the northern Mexican Pacific, Gulf of California, and Mexican Caribbean. These regions are priority marine ecosystems in Mexico, and the fisheries therein are of high economic value despite being of small scale. In all small-scale fisheries in these regions, fishing cooperatives, such as the ones included in this study, work to organize, manage, divide earnings, and make decisions.

The questions that guided this study were: What expressions of masculinity are found in fishing communities and how are women perceived? What are the implications of the participation of women within these fisheries? Could masculinity be a cause of risk-taking or accidents in fisheries? Have the expressions of masculinity in the fisheries changed over time? To answer these questions and account for the complexity of masculinity, (1) four expressions of masculinity were identified; (2) the involvement of women in decision-making and fisheries management was analyzed; (3) the risks associated with masculinity were documented; and (4) the benefits associated with changes in masculinities were investigated.

## Methods

**Fig. 1:**
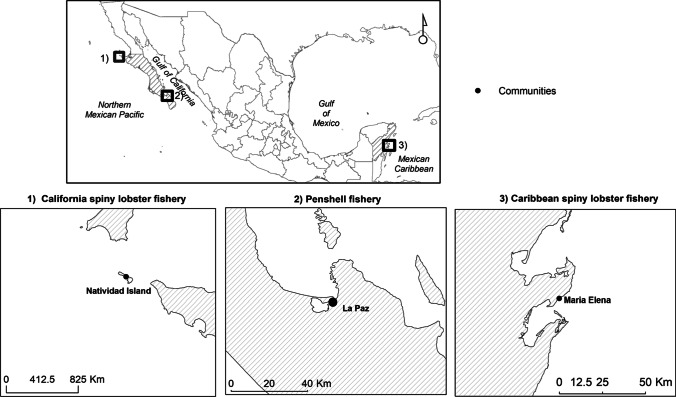
Geographical locations of the fishing communities where expressions of masculinity were characterized

Three fisheries were selected for this study: the California spiny lobster (*Panulirus interruptus*) fishery in the northern Mexican Pacific, the penshell (*Atrina maura*) fishery in the Gulf of California, and the Caribbean spiny lobster (*Panulirus argus*) fishery in the Mexican Caribbean (Fig. 1). These fisheries were selected because they were previously considered in another study (Solano et al. 2021), which assessed the involvement of women in fishing supply networks, thereby allowing for the results of the present study to be compared with the information generated in 2021.

The key informants were selected based on their relationships and life experiences in the community, cooperative, and fishing activities. Around 5–6 interviews per fishery were carefully conducted given the different perspectives among the informants regarding masculinity and work. In the qualitative methodology, it was important that the key informants have characteristics representative of a sociocultural group, accounting for the practices and meanings contained within the object of study (Flick 2004; Ito and Vargas Núñez 2005; Kvale 2011). For this study, the informants are representative of a sociocultural group in which several generations of men and women have dedicated themselves to fishing as a way of life, with gender stereotypes ranging from the most traditional to those that integrate changing masculinities with pathways that lead towards gender equality, which turned out to be highly relevant for our research (Olavarría, 2013, p.7).

A total of sixteen semi-structured interviews were conducted from July to September 2020, with nine women and seven men that participated in different stages of the supply networks. Men held positions as board members, fishing partners, aspiring partners, lab workers, cooperative secretaries, and surveillance presidents, while women held positions as community leaders, processing plant employees, fishing partners, and housewives of fishers. Due to the variety of positions that the informants held, a broad and open perspective on life within three levels was generated based on the community, the fisheries, and the ways in which masculinities are involved in both. Given the ethical principles of social research and to safeguard the identities of the participants, their names and their regions of origin have been coded (Table 1). The code is composed of four characters indicating gender [H (male) or M (female)], region [PN (northern Mexican Pacific), CM (Mexican Caribbean), and GC (Gulf of California)], and interview order (Table 1).

**Table 1:** List of people interviewed. The region of origin, principal position, and unique identification code of each interviewee are shown

Region of origin	Identification code	Main activity
1 Northern Mexican Pacific	HPN1	Aspiring partner
	HPN2	Laboratory
	HPN3	Board member
	MPN1	Housewife
	MPN2	Community leader
	MPN3	Processing plant employee
2 Gulf of California	HGC1	Board member
	HGC2	Fishing partner
	MGC1	Partner
	MGC2	Board member
	MGC3	Surveillance
3 Mexican Caribbean	HCM1	Board member
	HCM2	Fishing partner
	MCM1	Housewife
	MCM2	Aspiring partner
	MCM3	Housewife

The initial “H” or “M” of each code refers to the sex of the person [H (male) or M (female)]. The following two letters indicate the region of origin: PN (Northern Mexican Pacific), CM (Mexican Caribbean), and GC (Gulf of California). The final number is an individual identifier, which was assigned sequentially for males and females

Due to the lockdown measures imposed by the COVID-19 pandemic, the interviews were conducted via video calls through the digital platforms of Zoom or WhatsApp. The interviews were scheduled during convenient times for the participants to promote the development of close relationships between the interviewers and interviewees. It was important to ensure these participants were interviewed because of the fundamental roles they play within their communities and in the decision-making of their respective fisheries.

The interviews were used to collect information on work, family, and community life and to discuss the expressions of masculinity in a gender-driven context. It was relevant to scrutinize the perspectives of the informants with regard to the expressions of masculinity and the difficulties associated with the participation of women in fishing activities. To achieve this, an interview script was developed for men while another was developed for women (Online resource 1). The scripts were created following a qualitative approach, and the main topics explored in the interviews were (1) expressions of masculinity; (2) the involvement of women in fisheries; (3) risk-taking, self-care, and the physical and emotional health of men in fisheries; and (4) the benefits associated with changes in masculinities. The number of questions per topic was balanced, and all categories were covered. However, each participant was allowed to narrate their experience in depth. The interviews lasted between 60 to 120 min and generally took place after working hours for the informants.

At the end of each interview, the information was transcribed and encoded using MAXQDA 2020 (VERBI Software 2019). Throughout the study, the ethical principles of respect, autonomy, confidentiality, the common good, and fairness towards the person being interviewed, their family, and their community were prioritized. Furthermore, every person consented to the interview and the privacy of their personal information. Individual consents are recorded in the audiovisual archives; these can be consulted upon request.

The analysis consisted of organizing the content, identifying meanings, and defining categories from the interviews and findings (Ito and Vargas Núñez 2005; Kvale 2011). Following the approach of Connell and Messerschmidt (2005), we have identified three main themes: the distinction between public and private; masculinity studies focused on occupational masculinities, which refer to the jobs associated with men; and the international agenda, as expressed in the commitments and objectives of gender perspectives and equality. The information from the participants was organized into four resulting expressions of masculinity.

## Results

### Expressions of masculinity in Mexican fishing communities

The results from the three interviews show that the expressions of masculinity can be characterized and grouped into four categories: (1) reluctant traditional masculinity, (2) flexible traditional masculinity, (3) transitional masculinity, and (4) apprentice masculinity. The four identified expressions of masculinity were defined from the data obtained in the interviews and were based off of the concepts of hegemonic masculinity proposed by Connell (1995), Connell and Messerschmidt (2005), Kaufman (1989), Kimmel (1997), and Núñez (2017). Considering that gender is constructed in practice and the different participation scenarios proposed by West and Zimmerman (1987), we adopted the concept of apprentice masculinities proposed by Lave and Wenger (1991). Twenty-two attributes encompass the different dimensions that define the expressions of masculinity, and their consistent presence in case studies is evidence of the regularity and stability of the categorization process. The characterizations of traditional reluctant, traditional flexible, and transitional masculinities and their main attributes are described in Table 2. Apprentice masculinities are not included in this table, as their characteristics may vary depending on the mentor and mentorship path.

**Table 2:** Expressions of masculinity

	Characterization of masculinities		
Principle characteristics	Reluctant traditional	Traditional flexible	Transitional
Born in a fishing community	X	X	X
Men over 50 years of age	X		
Men between 40 and 50 years of age		X	
Men between 30 and 40 years of age			X
Socialized in activities related to fishing	X	X	X
Endorsed by other experts	X	X	X
Body and mind used in the work of the fisher	X	X	X
A part of a fishing team or fishing cooperative	X	X	X
Fishing is the only work activity	X		
Established as a couple	X	X	X
They are considered the breadwinner and main family authority	X	X	
They consider that women’s activities should be exclusively domestic	X	X	
They manifest superiority towards women	X	X	
They are observed as superior to other men	X		
They are reluctant to change	X		
They take care of their health			X
They accept that reality has changed		X	X
Women are accepted into various fishery activities		X	X
They consider other life options outside the fishery			X
They educate their children to aspire to a life outside the fishery		X	X
They accept the empowerment of women			X
They try to be co-responsible within the home			X

The four expressions of masculinity were present in the communities of the Northern Mexican Pacific, Gulf of California, and Mexican Caribbean. This is largely due to the similarities among fishing activities and the processes of becoming a man in these three communities. However, the participants defined being a man according to the sociocultural context in which they found themselves and the region in which they worked. In other words, men can share the core category of reluctant traditional masculinity in the Northern Mexican Pacific, Gulf of California, or Mexican Caribbean with different manifestations (Table 3).

**Table 3:** Differences in the expressions of masculinity by region

Types of masculinity	Northern Mexican Pacific	Gulf of California	Mexican Caribbean
Reluctant traditional	They accept that women are a part of the cooperative as long as they dedicate themselves to “women's” tasks, such as administrative work	The imposition of the ban was not taken as being written in stone, as they had to support their families at any cost, which led many men to engage in illegal fishing. When they were told that women should join the cooperatives, they saw it as a requirement to continue operating during and after the ban	Fishing has been and will continue to be a means of supporting families in the Mexican Caribbean. There is a reluctance of women to participate in fisheries, as they consider the field to belong to men
Traditional flexible	They recognize that the work of women, such as oceanographic monitoring, is important to promote the development of the cooperatives	For them, staying exclusively at sea was not a viable option to support their family. Raising a family led them to accept that their wives engage in paid work activities	They have incorporated different ways of thinking, either because they have accepted the proposals of the young members of the association (e.g., attending gender equity courses) and accepted the incorporation of women into the fishery
Transitional	Changes in ideology stem from academic training on land, in addition to relationships with empowered women who have shown them that they should be co-responsible within the home	Changes in ideology stem from interactions with people from different contexts, from the self-imposed ban, and from relationships with women who, upon entering the workplace, have required co-responsibility at home	They consider women as partners both in the workplace and at home. They think that avoiding alcohol and taking care of their health will benefit the cooperative
Apprentice	They are young people who work with older and more experienced men. They were not born into a fishing family and came to the island to learn the trade	They enter the fishery as workers and their main aspiration is to become a fisher	They are generally accepted by veterans as workers

The expressions of masculinity in the three regions indicate that fishing has been historically perceived as a job for men. These expressions are present in the social imagination shared by men and women in each region and in the everyday activities used to extract marine resources, which provide recognition and economic and social value, as mentioned by HCM1.

“There are many customs [and] traditions. Almost 80% of the population are fishers—parents, grandparents, uncles, [and] nephews—and for anyone to learn to be a fisher, we have to take them fishing.”

Masculinities and fishing form an indisputable continuum, and fishing activities are learned through practice (Piñeiro et al. 2016). Learning takes place daily among family and friends and is complemented by working with other fishers.

“The people teach you how to work [...]. It is like a little school that follows you around each day, and it is a daily routine, so everyone knows their jobs; a chain is followed [...]. You learn until you gain confidence and start working.” (HPN3)

Gender is incorporated into fishing activities, with men being assigned extractive activities because of the inherent difficulty and risks of working at sea. In other words, a masculine identity is built on the activities of the fishery, considering that the lives of men revolve around fishing. This is learned from a very early age. Boys grow up with the idea that one day they will be recognized as seamen, fishers, and members of a cooperative, to which they will need to dedicate a large part of their lives to achieve success.

Being a member of the fishing sector implies continuous learning of what each person can and should do as they gain expertise and recognition. What constitutes being a woman or a man in the fishery is established through socialization. For the men of the California spiny lobster fishery, the penshell fishery, and the Caribbean spiny lobster fishery, dedicating themselves to the fishery is a decision that is shaped by two elements: a need for work and a fondness for the sea. These elements are particularly present in older men, who transmit this perspective from generation to generation despite the risks associated with fishing. For instance, one of the informants (HGC1) commented that they have been learning since they were a child, and that they are now part of all those who have preceded them.

Belonging to a line of fishers shapes male identity and life expectations and constructs the collective ideal that many people hold in small-scale fisheries. Being part of a team in a fishery provides social and symbolic value for men, in addition to providing them with relatively good working conditions, incomes, and social status with the expectation of becoming partners in the cooperative.

Doing the job well is in part the result of a transference of knowledge from other experts, boatmen, divers, members of academia, and civil society organizations (CSOs). In all three fisheries evaluated in this study, working hard and demonstrating a clear understanding of how to do a job well were considered essential for a person to be able to call themselves both a man and a fisher, regardless of the hours invested or the associated risks. For example, in the Gulf of California, a decline in penshell abundance led the community to implement a fishing ban. This allowed for damaging practices and behaviors to be identified, and many men had to learn to take care of this resource while redefining their ideas (Solano et al. 2021).

A person can strive to be a man by demonstrating that they are a hard-working team player that labors for the good of the community. “A hard-working man is welcomed by everyone because as a hard-working man, he gets along with everyone and refuses absolutely nothing, whether he is a partner or an intern” (HGC1). Being a man also means living responsibly. The interviewee HCM1 said, “You are not a man when you are overly relaxed, when there is no seriousness.” Thus, becoming a partner in a cooperative implies that an individual has fulfilled their work responsibilities in the fishery, has formed a family, and is recognized and valued by fellow community members.

In these scenarios, the expressions of masculinity take shape as something embodied not only by men but also by the community as a whole, including women, children, and teenagers. The differences within the expressions of masculinities will be explained in further detail in the following sections.

#### Reluctant traditional masculinity

Reluctant traditional masculinity was generally identified in men over 50 years of age. These men consider that in addition to being fishers and members of a cooperative, they must also form a family with a wife who will always accompany them without ever neglecting their home. These men consider that fishing will always constitute the sole means to support their families. This coincides with a gender-based division of labor, with men acting as the exclusive providers and their wives acting as their counterparts in the domestic sphere. Women, from this expression of masculinity, oversee cleaning, raising children, cooking, and ensuring everything is ready for the fishers to fulfill their duties.

The notion that men should focus on work while women stay at home stems from the historical division between public and private spheres. In this context, men obtain goods through their physical strength, which they employ to extract marine resources, and believe that women should receive and care for them at home. Reluctant traditional masculinity is a result of what men and women have learned about gender from an early age.

“Well, most of the women are older, and their mindset has been present since they were little, which is to take care of their home. Once they got married, [their husbands] set the limit of ‘you take care of the home; [you] take care of the children, and I'm the one who brings the money.’ Then [the women] are surprised, and it’s like they do not even want to help a woman get ahead.” (MCM3).

As the main responsibility of these men is their work, they are generally satisfied by what they do. These men are used to conducting the same daily routine. Thus, it is difficult for them to adapt to new scenarios, and they resist change. This impacts their relationships with others; they show a sense of superiority, as shared by the following interviewee.

“Let’s just say there are many people, fishers, who are like this. How do you say it? They are old-fashioned; the man is in charge; the man says what has to be done; many people here still have this deeply rooted [within themselves].” (HGC1).

These men think traditionally, and they are reluctant to change, even when the change is beneficial. The interviewees considered that this group of men can affect the development of a cooperative because they limit progress by not wanting to change their way of thinking.

“Here in the fishing cooperative, the main issue deals with innovation. They [reluctant traditional masculinities] do not want to change their ways and how they work, even when there is a much better plan. This causes good opportunities to be lost! Also, this prevents the cooperative from moving forward. These workers limit themselves with this mentality. I think they are afraid of things; they are scared to change what is traditional when they can join the traditional together with the new and generate good results.” (HCM2)

In addition to this, a reluctance for women to participate in fisheries is present, as these men consider fishing to be exclusive to men. The gender-based division of labor is perpetuated and has such deep roots that it is almost considered through a naturalized viewpoint, as if it has always been there. When men with reluctant traditional masculinities were told that women should join the workforce, embarking down the path to gender equality became a requirement for the cooperative to continue operating.

Some women face scrutiny and criticism for daring to break assigned gender norms. These women have managed to leave home to engage in paid activities, although they employ double shifts to demonstrate that they can handle their responsibilities at home and their work without the support of their partner. For reluctant traditional men and some women in the communities, their work is minimized and irrelevant.

“I have noticed something in my time here in the organization: that many of them [reluctant traditional masculinities] have not changed their way of thinking, and I find it very difficult for them to do so. It is hard for them to accept that women are now part of the cooperative, that we are their partners. It is as if they have not realized what we have achieved or that we are participating in fishing activities and all that; they are not pleased by it. They already have erroneous ideas—they get them from their ancestors, from a long time ago—that the woman is always at home and the man at work.” (MCM3).

It is important to recognize that there are two diver positions within fishing communities: fishing diver and scientific survey diver. Scientific survey diving is not easily compared to extraction diving. This kind of diving highlights the need for a cooperative to collect data to make the best decisions about its fishery. Scientific survey diving is an activity in which women have become involved; however, some men with reluctant traditional masculinity are upset by this and other ways young women are becoming involved in fisheries (Torre et al. 2019). They consider that these women are stealing spaces from other men who have been waiting for years. They believe that women entered the fisheries without having to go through the entire process required to attain the position in question. These men also consider that due to their physical makeup, women cannot do some jobs:

“They [women] want to go to work without climbing the ladder because when you come to work here, you come from the bottom, right? You arrive cleaning, right? You arrive driving the car, loading bait, loading gasoline, and obviously, you cannot send a woman to carry those things; they are very heavy! So, you cannot send a woman to dive because you would be giving her 3 or 4 years of advantage over a man, and these women would not be willing to do that.” (HGC2).

The men with reluctant traditional masculinity did not agree with women being integrated into these spaces in part because they see this as an unfair advantage but also because of the risks posed by fishing or surveillance activities. These activities are conducted through beach patrols to ensure the catch is not stolen. Risk, in this line of thought, is associated with masculinity; thus, these men prevent and hinder women from participating in fisheries. They accept women in the cooperative so long as they dedicate themselves to what they call “women's work.” For example, women may be secretaries but not divers, which blocks the development and participation of women in fisheries.

The results indicate a close relationship between the expressions of gender in fisheries and being a man. Inside of reluctant traditional masculinity, physical and emotional care is not considered part of being either a man or a fisher. This traditional masculinity puts the physical and emotional health of some men at risk, silencing or masking their discomfort. The risk behaviors of the men in these regions were related to seeking out recognition through their work without considering the implications for their health. Even when the risks are recognized, they are not discussed among fishers, as the commitment to not abandon or let the team down is of upmost importance. This makes these men reliable workers, which in turn allows them to be considered, both by themselves and their peers, as both good men and good fishers.

“… as a man, one is more stubborn, right? In other words, a man wants to keep working, keep giving his all. It’s okay! When you’re young, you don’t care about the rest, but what you want is to work, work, work.” (HPN3).

Reluctant traditional masculinity encompasses not only the identity of a man but also his approach to relationships, learning, and working. For these men, providing for their families is crucial because it is directly connected to being a man. They consider the work as a gendered space, with their positions being gained through time and effort. Women are viewed as not able to fulfill their duties, as they did not start from the bottom. Thus, they are not up to the tasks at hand and for this reason, women have limited access to manage fishery resources. However, these men are also the ones who are most negligent with their health and self-care. They engage in risky activities without considering how it will affect people that may depend on them. This expression of masculinity is deeply ingrained into fishery practices, as these men are prominent members of the community. Some men who have distanced themselves from this expression of masculinity while not entirely abandoning their traditional viewpoints become flexible and open in certain ways.

#### Flexible traditional masculinity

Men with flexible traditional masculinity are between 40 and 50 years old, and their characterization and expression of masculinity includes being a fisher, a member of a cooperative, and forming a family. Their wives may be women who engage in economic activities outside the home but without neglecting household duties. These men have incorporated flexible thinking, accepting proposals from young staff or cooperative members to attend gender equality courses. These men acknowledge the importance of incorporating women into fisheries and supply network activities beyond those of administrative work. They recognize that it is important for the development of the cooperative for women to participate in fishing activities, such as oceanographic monitoring or surveillance work.

Some men were in the process of changing their reluctant traditional thinking for more flexible thinking by accepting that their wives engage in paid activities outside the home and that they should participate and be included in the same cooperatives.

“… [She] did not ask me for permission! … We all had to work. Women and men work here in the community equally; there are surveillance women. My wife is one of them, in fact. She is currently surveilling; we perform equally ...” (HGC1).

It has been a struggle to achieve recognition for the participation of women in fisheries. Some men have come to change their minds after their wives found paid work because the income they themselves provided was insufficient. At first, these men were reluctant to accept that their wives worked and earned incomes, viewing this as an attack on their masculinity. As one of the women interviewed (MGC3) pointed out, “[my husband] did not accept it, he asked me who was going to clean? Who was going to make the food? Who was going to take care of the children?” When this woman shared the benefits of her work with her family, her husband began to change his mind.

For some people, this is *machismo*, and they note that it is rooted in the oldest people.

Men with flexible traditional masculinity have learned to work with women in some areas of the fisheries, and they have changed their thinking and positions, acknowledging that women can also carry out important activities in the supply networks. However, these men continue to consider that household activities are the sole responsibility of women. Although their wives have paid work and are part of the cooperative, they are still responsible for domestic activities. Women manage the responsibility of performing household duties like preparing food or cleaning the house by either doing the work themselves or by negotiating with other women in the family (e.g., sisters or sisters-in-law).

While flexible traditional men are willing to accept the presence of women at work, they are still struggling with accepting the risks that come with being a fisher. Risks are as much a part of fishing as the weather or the sea, yet others depend on the care that men take while fishing. The testimony of HCM2 describes various situations in which the *machismo* of some men is apparent when they go diving and risk their lives. These behaviors are learned from traditional masculinity models.

“I just think it is stubbornness because they have taken courses, but these are stubborn people, and they do not want to stop risking their lives. It is stubbornness because there are times when they compete to see who dives the deepest to show how *macho* they are ... it is an ingrained mentality that has been present for many years and that has never left. Unfortunately, they pass it on to young divers. So, this mentality does not allow them to take precautions so that they do not risk their lives!” (HCM2).

Other health risks are related to stress and tension. When fishing bans are established, many men suffer damage to their physical and emotional health, leading some to engage in risky activities. Interviewee MGC2 describes this situation: “My husband worked at night and had an accident. He suffered very bad decompression sickness from going to work at night, from going to work illegally. He fished with a harpoon. He no longer looks good; he no longer dives!”.

Most of the interviewees consider that accidents and various health problems are inherent to being a man. Traditional masculinity is considered *machismo*. This puts not only the physical health of men at risk but also the stability of their families by threatening their incomes. This particular way of expressing masculinity entails behaviors that have consequences for other people, not only the men themselves.

Rejection, a lack of acceptance, and the exclusion of the participation of women in fisheries are the result of thought processes centered on traditional masculinities that resist change. Understanding the value dimensions of various forms of work and recognizing the usefulness of monitoring and surveillance activities will lead to a greater acceptance and recognition of women in fisheries. It is relevant to mention that these men embrace the possibility of women working in fisheries, although this is inspired by necessity or changes in broader contexts.

Flexible traditional men are open to working alongside women, yet they still espouse attitudes, behaviors, and ideas associated with reluctant traditional masculinity, such as substance use, a disregard for their health and well-being, and a continuous exposure to risk. The transformation away from this expression of masculinity is steady and slow yet unavoidable as men discover they are immersed within bigger contexts, such as the cooperative.

#### Transitional masculinity

Transitional masculinity was identified in men between 30 and 40 years old. These men were characterized by holding roles within the supply networks that were not those of a fisher. These men were mainly the children of members in a cooperative or people who have returned to their communities after studying elsewhere because they do not see themselves living outside of the fishery. Many of the changes in the way these men think come from academic training on land, interactions with people from different backgrounds, and the relationships with the women they went to school with who have subsequently made decisions about their lives when entering the workforce. These men consider that the roles of women can be any that women want. These men have incorporated new ways of thinking about gender equality because they have attended training courses that have provoked reflection, which has led them to assume more egalitarian and equitable positions in relation to women. These men consider women to be their partners in the workplace, as expressed by HGC1. “Women are also very capable of doing any type of work inside the fishery.”

In younger generations, viewpoints are changing. These men are more flexible and view women who work in the fishery or cooperative favorably and are characterized by flexible traditional masculinity, realizing and recognizing that the work of women benefits themselves and the entire community. Some interviewees commented the following:

“... the women who work diving do us good; they are dedicated to conservation and to all those kinds of activities that support the community; as women, they will always have my support!” (HGC1).

“… I have never had problems with women participation. In fact, in this case, women divers are my partners because we also took the diving course and we automatically became partners, right? I have dived with them. For me, it is very cool that they are involved in some way.” (HGC3).

Understanding the value dimensions of the work inside the fishery and highlighting the usefulness of monitoring will lead to a greater acceptance of the participation of women in fisheries due to a recognition of their activities, as an interviewee pointed out:

“…because for many, it does not work; the work they do makes no sense. Why? Because they do not see it reflected; they do not see that the divers go under and then there are more abalone or snails. So, many people do not see the causes of things, of the actions of the girls, and since they do not see them, they cannot say that what they are doing is very valuable. What they are doing is very important for the company!” (HPN2).

When it is possible to move from a masculinity of traditional reluctance to traditional flexibility, future generations will have the possibility of living with transitional masculinity. Under this form of masculinity, men and women can live together in families and work together without stereotypes, which will encourage women to participate to a greater extent in fisheries with increased recognition for their work. The reflections and conclusions generated in gender equality workshops that have been conducted by COBI have been able to make some viewpoints more flexible, which serve as examples for other cooperatives. The members of other cooperatives observed the benefits that come from incorporating women into their fisheries, as described by the interviewee MGC3 when retelling how safe areas have been once again established thanks to the surveillance efforts carried out by women.

Just as some men are more flexible in their way of thinking, others have gone further, moving towards the incorporation of more equitable actions. One more element to consider is the possibility for development, both personal and in the workplace, as described by the interviewee HCM2: “…between the two of us, we have tried to carry the responsibilities of our household.”

A final element of transitional masculinity that should be recovered is the possibility for women to make decisions about their lives. For this, it is necessary to generate more egalitarian spaces for relationships. This would be beneficial for cooperative members, families, and communities.

Men with transitional masculinity spend more time with their families, share household activities, and take care of their children, as women can now participate in paid activities outside of the home. This gives an important weight to their paternity, and many are educating their children to have a profession beyond the fishery. These men avoid abusing alcohol and actively take care of themselves, which improves the cooperative.

“… I really want my child to be in the place that many have told me that I should be, which is a place higher than mine. Do I want him to support fishers? Yes, of course I do, but in a professional way. I say this because I would like him to grow [into positions] much higher than [mine], for him to reach where I could not go, and I know he will succeed!” (HGC2).

The results allowed us to identify that equality implies a recognition and acceptance of difference and not the exercise of power and competition between genders. Regarding health conditions, some benefits were identified in the transformation of traditional reluctant masculinities to flexible ones. One interviewee refers to training and learning to minimize the risks associated with diving.

“People who have worked with COBI, who have been following the protocols that they use in diving— the times, the dives—they are very prudent. They take the time to make safety stops, [keep track of] the time they are down, [and] take care of the weights that are dragging them down. These are the practices that have been least affected and that encourage other colleagues to do the same.” (HPN2)

It is important to emphasize that change generates benefits by reducing risks, allowing for improved organization and encouraging the joint, non-competitive participation of men and women to obtain mutual benefits. If this is not seen as competition but a process to improve working and living conditions, men will allow their thought processes and masculinities to become more flexible for the good of the cooperative, the community, and themselves. Transitional masculinities are the result of learning and improving outside the community and then returning and transforming gender dynamics. Another characteristic of this expression of masculinity is that after having taken courses, men may incorporate them into their practice as fishers, even if they have lived inside the community their whole lives.

#### Apprentice masculinity

Young men between 20 and 30 years of age who have recently entered the fishery as workers are characterized by apprentice masculinity. These men are referred to as “apprentices” because they learn their trade, identity, social values, and roles as men. In other words, there is a situated learning process that is based on the mentor and the apprentice.

Men with apprentice masculinity work with the older and more experienced men. Their main aspirations are to be responsible and committed so that they can become members of a cooperative. A long process is required to become a cooperative member, so men with apprentice masculinity try to be “aspiring partners” first and then try to rise in position. Differences in their way of thinking depend to a large extent on the expression of masculinity exercised by their mentor. Hence, some apprentices behave in a macho manner, endorsing stereotypes of reluctant traditional masculinity. Yet, others show greater acceptance for the participation of women in the supply networks, with more flexible thinking that incorporates the principles of gender equality.

Learning to become a fisher involves introducing the “aspiring partner” into the community, having a mentor, knowing the craft, embodying the values, and being validated by others. This learning takes place within the context of the fisheries and is experienced by doing. As stated earlier, apprentices often start from the bottom, loading the bait and cleaning boats. It should be noted that this is an expression of masculinity that is being formed.

The references or models from which men with apprentice masculinity extract their attitudes, beliefs, standards, and attributes relate to the mentor who oversees them. Each day they work for a place in the fishery but also to become a certain type of man.

“For young people, the fishery is the only thing there is. They are very attached to that, and they don't really see any other alternative than the sea in this company [...]. Then, the mission for a man is to be fully in charge of his family while at sea.” (HPN2).

The way in which these men consider the participation of women in fisheries largely depends on their learning process:

“It [makes me] proud that women can now do the work that men have done in fishing. With gender equality, anyone can do any job.” (MCM2).

However, other young apprentices might not agree with women doing work inside the fisheries. Further, apprentices may engage in risky behaviors, which is part of the inheritance of traditional masculinity that is incorporated by young fishers. This includes the consumption of alcohol and other drugs, which are prohibited and monitored by the cooperative yet remain an open secret. “Alcohol is unfortunately very harmful for them. The vast majority drink and that increases the risk that something could happen to them at sea. They have already had bad experiences, unfortunately.” (HCM2).

Men with apprentice masculinity also acknowledge the risks of becoming a fisher, although how they handle these and incorporate them into their expression of gender remains to be seen. This also begs the question of the value that they will place on family and whether or not they will take care of their health and overwork themselves. The benefits of the changes due to this expression of masculinity will be noted in the future.

## Discussion

The results of the study from the three fishing communities in Mexico show that the participants defined being a man according to the context in which they found themselves. These expressions of masculinity incorporate four types for each region: (1) reluctant traditional masculinity, (2) flexible traditional masculinity, (3) transitional masculinity, and (4) apprentice masculinity. These categories are not static and represent different approaches to being a man and largely reflect a gender culture that is based on inequality and that impacts living conditions in both public and private spheres (Kabeer 1998; López and Bradley 2017; Castañeda Camey et al. 2020). Although this culture is changing and moving forward, there is still a deep commitment to old ways of thinking and traditions.

According to the approach of the United States Agency for International Development, inequalities can be considered an act of gender violence based on the impacts on the lives of women, families, and communities and consequently global development (USAID 2012). In the case of small-scale fisheries, women are undervalued or excluded in decision-making (WorldFish Center 2010). Even when women show broad participation in pre- and post-production activities (World Bank 2012; FAO 2020; Harper et al. 2013; Teh and Sumaila 2013), they are often limited to these activities in fisheries management organizations like cooperatives given that fishing privileges have historically been granted to men (Castañeda Camey et al. 2020; Solano et al. 2021), which represents an inequality.

There is a reluctance for women to be incorporated into these fisheries because women are not considered to be sufficiently skilled or able to put up with the demands of the profession. Inequalities regarding the participation of women in fisheries and decision-making are related to traditional perspectives on fishing, which is viewed as an activity for men and associated with the bodily attributes of strength, toughness, and endurance (Adkins 2010; Turgo 2014; Salguero and Alvarado 2017; Castañeda Camey et al. 2020). Fishing is synonymous with masculinity, and this perception is generationally transmitted in Mexican fishing communities (Toledo 1999; Aguirre et al. 2014; Padilla and Perez Lanza 2014; Siegelman et al. 2019). A metaphor proposed by Alonso-Población and Niehof (2019) links women with the land and men with the sea, which reflects the normativity of the cultural models that structure the gender-based division of labor in fisheries. This division is found in activities associated with processing and product commercialization and is also found in family traditions and fishing communities. By not considering the contributions and importance of women in fisheries, an ideological connection between fishing and capturing resources is reinforced, which in turn further prevents women from exercising their rights as fishers.

Gender relations shape interactions and incorporate elements of dependence, control, and submission in a feedback system at work between the public and private spheres, which has become naturalized due to the resulting gender expectations and stereotypes (West and Zimmerman 1987; Conway et al. 1996) that inevitably limit opportunities for development. Social and economic valuations differ between home and work, with traditional expressions that are detrimental for both men and women, especially regarding health given that men often partake in risky behaviors at work and consume alcohol and drugs (García 2005; Tu-Anh Hoang et al. 2013; Turgo 2014; Coulthard et al. 2020). Negative masculinity (García 2005) can also develop, which is a product of gender learning in men (Salguero et al. 2018). In this study, we could observe that reluctant traditional men have naturalized the notion that they represent these values linked with manhood, money, and power, among others.

However, the results of this study also showed some changes in expressions of masculinity and flexible thinking processes, indicating a move towards new relationship forms and the acceptance and recognition of the participation of women in fisheries. It was possible to identify men who have changed their way of thinking while taking care of their health and avoiding risky behaviors. The passage from reluctant traditional masculinities and flexible traditional masculinity is encountered with challenges and defiance. For instance, women going to work is not always viewed with ease and comfort. Men with transitional masculinity carry the potential for change. These men tended towards equality by distancing themselves from the hegemonic model of masculinity. This is consistent with the results of other recent studies that have highlighted possible changes in masculinities (Pini and Conway 2017; Gustavsson and Riley 2018, 2019; Coulthard et al. 2020).

It is necessary to de-essentialize how men are conceptualized and to view them as gender subjects in specific social practices (Connell 1995; Núñez 2017). The results of this study coincide with those of Gustavsson and Riley (2019) regarding the relational character of fishing masculinities, incorporating the experiences of young fishers with those of their mentors in addition to changes associated with family life and income. New practices can be incorporated into more traditional aspects of masculinity. In this sense, the approach of Connell and Messerschmidt (2005) is retaken in terms of masculinity not being a “fixed entity embedded in the body or in the personal traits of individuals” but rather a “configuration of practices” that is achieved through social contexts.

Tools, such as gender equality courses, provide men with the opportunity to reflect and unlearn some of their attitudes and behaviors. When viewed within the ever-changing context in which newer generations and women demand equitable conditions and for women to be incorporated into fisheries, it becomes evident that men are not fixed in historical contexts. Their identities are always changing because the world around them is always changing. Men in fisheries display gender cultures that incorporate force, rudeness, and resistance, but workshops on gender equality and changes in the ways in which women operate within their families and fisheries supply networks have encouraged men to develop other ways of “being a man” with more flexible thinking. As Filteau (2014) points out, not all men legitimize unequal gender relations, nor do they all display traditional hegemonic masculinities. Masculinities are not static and are open to change (Hopkins and Gorman-Murray 2014). That is, expressions of masculinity are geographically, culturally, and temporally defined (Gustavsson and Riley 2019) and can be redefined over time given sociocultural changes as well as structural changes in the fishing industry.

In this sense, a possibility for positive change is present since the data indicated flexible ways that masculinities may be recast. Accepting new values and practices regarding masculinity is important for both men and women. The construction and promotion of a positive and non-violent version of masculinity requires that both men and women possess the necessary knowledge, skills, advice, and the support of their peers, associates, and cooperatives (Tu-Anh et al. 2013; García 2005). Fostering well-being through awareness workshops will generate opportunities for men to reflect on gender equality with others in their communities (UNDP 2000). This constitutes an important goal of international and developmental agencies, governments, and CSOs. Women and men are immersed in a relational context. For the former to be included in gender-based jobs like fishing, the latter must play very important roles. In this regard, men hold the possibility of change, as they are the ones in positions of power, acting as decision makers and community leaders.

## Conclusions

This study found four expressions of masculinity in fishing communities that limited or facilitated the inclusion of women in fisheries. Older fishers were more reluctant to change their vision of gender equality. As such, their involvement in reflective processes and activities can be sought out, but the primary efforts should be aimed at facilitating change in the younger generations. Younger generations can be guided to build more equitable and egalitarian expressions of masculinity. Bringing apprentice masculinities closer to transitional masculinities will help young people to be raised under the principles of gender equality.

This proposal can be implemented in multiple ways if we consider that men with apprentice masculinity are still forming their identities and finding ways to contribute to the community. Thus, it is necessary to promote strategies that young people may employ to avoid replicating harmful traditional patterns while transitioning towards more egalitarian practices and co-responsibility, even when caring for themselves, in which men and women have the same development opportunities.

Awareness training workshops will allow for the development of reflective and flexible thinking aimed at supporting the transition to gender equality. At the same time, these workshops can help to incorporate a new perspective of the future in young apprentices in which traditional masculinities are replaced with more egalitarian forms. This will benefit cooperatives, families, and the men themselves, as the importance of health, self-care, and avoiding risks may be promoted.

It is important to work with reluctant traditional men to allow them to think differently and embrace new ways of working by incorporating women into fisheries and recognizing their contributions. In turn, it is important for these men to realize that the ways in which they learned to be men and fishers has encouraged them to assume risky behaviors inside and outside of work (e.g., drugs and alcohol consumption) and to avoid responding to the demands of their bodies given the physical demands of their work. These men would risk not only their physical health but also their mental health and those of their families, cooperatives, and communities.

These actions represent another reason to develop proposals for transformations based on reflective processes that include men and women engaged in reluctant or flexible traditional masculinities that have not moved beyond acknowledging gender differences. Strategies are needed for older and younger men to avoid replicating harmful traditional patterns while transitioning towards establishing equal opportunities and shared responsibilities between men and women.

Knowledge is necessary for complying with institutional criteria for gender equality while meeting the objectives of the 2030 SDGs (ONU 2015) and the Voluntary Guidelines for Securing Sustainable Small-Scale Fisheries in the Context of Food Security and Poverty Eradication (FAO 2015). Both international instruments act synergistically to achieve social, economic, and environmental sustainability and are related to one another via SDG1 (No Poverty), SDG 8 (Decent Work and Economic Growth), and the FAO guideline “Social development, employment, and decent work” by recognizing the roles of women in fisheries and in their household incomes, which support poverty reduction. In addition, SDG 5 (Gender Equality) and the “Gender equality” FAO guideline promote gender equality across the community and fisheries activities, while SDG 13 (Climate Action) and the “Disaster risks and climate change” guideline can be promoted through the contributions of women for climate action. Finally, SDG 14 (Life Below Water) and the guideline “Governance of tenure in small-scale fisheries and resource management” can be used to promote the responsible use and management of marine resources through the joint efforts of men and women.

International commitments to these criteria could be promoted by working with men with apprentice masculinity and continuing to provide tools through workshops that focus on reluctant traditional masculinities. The goal is to be rid of inequality and for women to be included in different contexts. In this case, fisheries represent a perfect opportunity to reduce poverty, promote gender equality, and preserve natural resources. This study may be a starting point to connect the international agenda of gender equality with specific locations and communities.

## Supplementary Information

Below is the link to the electronic supplementary material.

Supplementary file1 (DOCX 26 KB)

## Data Availability

Data is available upon request from the corresponding author, without reservation and to ensure transparency.
