# Supplementary material for: Characterization of masculinity expressions and their influence on the participation of women in Mexican small-scale fisheries
Source: Marit Stud. 2022 Jul 21;21(3):363–78. doi: 10.1007/s40152-022-00276-z (PMC9299965; doi:10.1007/s40152-022-00276-z)
Supplement: Supplementary file 1 — Supplementary file1 (DOCX 26 KB) [file 40152_2022_276_MOESM1_ESM.docx]

**Characterization of masculinity expressions and their influence on the participation of women in Mexican small-scale fisheries**

**Supplementary Material 1.**

**Interview script from men from the fishing communities**

Complementary material number one corresponds to the scripts used to carry out the semi-structured interviews. An interview script was designed for men and another was designed for women. The thematic axes to be developed were masculinities, the possibility of involving women in decision-making and fisheries management, shared family-work responsibilities, physical and emotional health, and changes in masculinities and within the fisheries. The number of questions per thematic axis was balanced; however, each participant was allowed to narrate their experience. The interviews lasted between 1–2 hours and generally took place after working hours.

**1) Masculinities**

1.1 Don [Name of interviewee]: Tell me about a day in your life.

1.2 And one day at work? Could you tell me how that goes ...?

1.3 How did you get into fishing? How did you begin? Tell me what you remember.

1.4 Did someone guide or teach you? Maybe your Father, Grandfather, or someone else?

1.5 Is fishing work important to men in your community?

1.6 And for you, what does your work represent? What do you feel when you go fishing? [Explore managing emotions and feelings in men]

1.7 What does it mean to be a man in your community?

1.8 What does it mean to be a man in your fishing organization?

1.9 What if your son does not want to be a fisher and wants to be a clerk instead? Or, to propose an extreme example, a dancer or some other job that is normally “reserved for women”?

**1) Masculinity and possibilities for the involvement of women in decision-making and in fisheries management**

2.1 Family-work

2.1.1 What does your work represent for the family?

2.1.2 How do your wife and children see you?

2.1.3 How did you organize the division of activities between you and your wife [You and her at work? Any sons or daughters?]

2.1.4 Does your wife and/or children participate in activities related to fishing or fisheries? Where do you normally perform these activities?

2.1.5 Have you had any difficulties, for example, in agreeing to make decisions about what each of the family members should do? How did you solve the problem?

2.2. The community's perception of the participation of women in fishing

2.2.1 What comments do community members make about women participating in fishing?

2.2.2 How does the community see women who are dedicated to conservation, for example, the women in the monitoring group?

**1) The risks of masculinity for the physical and emotional health of men**

3.1 Don [Name of interviewee]: Does the work you do fishing involve health risks?

3.2 Don [Name of interviewee]: Have you heard of any accidents or illnesses in the community due to fishing?

3.3 In what type of activity? Diving? Line fishing or with traps?

3.4 How did they solve it? Did they go to the doctor, take medicine, or take care of themselves?

3.4 Have you gotten sick from something? Gone to the doctor? What does your family tell you?

Emotional health

3.5 Don [Name of interviewee]: When have you felt sad? What did you do? Who did you tell or did you keep it in?

3.6 And when you are very angry, how do you express it? Do you yell, hit, leave, stay quiet, or do you hold on? Do you take it out on anyone? [Explore emotional management]

3.7 Don [Name of interviewee]: Have you heard if men in the community drink and has it caused them problems? What do they do about it?

3.8 Don [Name of interviewee]: Do you know of any men who use drugs in the community?

**1) Changes and continuities in fishing activities**

4.1 Looking back: What was fishing work like for your parents or grandparents and how is it now different for you or for your children?

4.2 What do you think has remained the same?

4.3 What things have changed?

4.4 Are there any things that have changed for the better?

4.5 Well, everything changes and change may not necessarily be bad, but what do you think?

WE THANK YOUR PARTICIPATION, YOUR TIME, YOUR WISDOM ...

**Script interviews women from fishing communities**

1. **Masculinities**

1.1 Doña [Name of the interviewee]: Tell me about a day in your life.

1.2 And one day at work? Could you tell me how that goes ...?

1.3 How did you start working? [Depending on the answer, was it was before you had a partner, got married, or lived together or was it after your relationship with your partner ended?]

1.4 Did someone teach you? Maybe your Father (Mother), Grandfather (Grandmother), or another man or woman? [Take care that this is not only applicable to those who participate in extraction activities.]

1.5 Did you face any difficulties? [With who? Your family, husband / partner, the director, or someone else?]

1.6 What do people in the community tell you about your work (paid or otherwise)? [Explore the points of view of others about the work, regardless of whether there any difficulties are present]

1.7 How does it feel? And what have you done about it [Do you remain silent, cry without being seen, answer and defend your place or your work, or something else?]?

1.8 Is the fishery important to the women in your community?

1.9 And for you, is your work important to you? How do you feel when you work? [Explore management of emotions and feelings in women when faced with work activities]

1.10 Are there women members in the cooperative? [If the answer is NO, why?]

1.11 Do you know what the process to become a partner entails?

1.12 Have you tried to become a member of the cooperative or would you to? [If you have tried and failed, why do you think that was? If you have tried and succeeded, what was your experience? If you have not tried, but would like to, what stops you?]

1.13 What does it mean for women from the community to participate in the cooperative?

1.14 What are the advantages of women working and actively participating in the cooperative?

1.15 What are the disadvantages of women working or participating in the cooperative?

1.16 What does it mean to be a woman in your community?

**2) Masculinity and possibilities for the involvement of women in decision-making and fisheries management**

2.1 Family-work

2.1.1 Doña [Name of the interviewee]: What does your work mean for your family? [Is it important, visible or invisible, or troublesome?]

2.1.2 How do your husband and children see you?

2.1.3 How did you organize the division of activities between your husband and yourself? [Does he work out of the house and you at home? Do your sons or daughters (apart from their work) do housework? Do you take care of your children, their education, or someone else in the house, including older adults?]

2.1.4 Do your husband and/or children participate in activities related to fishing or fishing? Where do they normally do these activities?

2.1.5 Have you had any difficulties agreeing when make decisions about what each family member should do? How did you solve the problems?

2.1.6 How does the community see the participation of women in the fishery?

2.1.7 How does the community see women who are dedicated to conservation, for example, those in the monitoring group?

2.1.6 Doña [Name of the interviewee]: Do you have children and are you engaged in fishing?

2.1.8 Doña [Name of the interviewee]: What would happen if your son/daughter does not want to be a fisher and wants to be a clerk or wants to dedicate themselves to something else?

2.1.9 [Name of the interviewee]: Thinking back, what was work like for your parents or your grandparents? How it is now for you? Do you think there have been any changes? Are they for the better or not so much? What do you think?

**2) Physical and emotional health care practices**

3.1 Doña [Name of the interviewee]: Does the work conducted by women in your community involve risks to their health? If so, which ones?

3.2 [Name of the interviewee]: Do you know of any woman who has become ill due to the type of work that she does in the community?

3.3 How did they solve it? Do they go to the doctor, take medications, or take care of it themselves?

3.4 Have you gotten sick from something? Do you go the doctor? What happened? What does your family tell you?

3.5 (To explore emotional care in women) [Name of the interviewee]: Have you felt sad, angry, very angry, or distraught? Why? What do you do when you feel that way? Do you tell your *comadres* or your family? Can you hold on? Are you taking it out on someone?

3.6 Lastly, Doña [Name of the interviewee]: Do you think that some women in your community solve their problems in a healthy way? Do any of them consume alcohol or drugs to solve their problems? What about the men in your community?

THANK YOU VERY MUCH, FOR YOUR TIME, YOUR DISPOSITION, YOUR WISDOM ...
